# Validation of biomarker-based stratification for risk of long-term outcomes after acute kidney injury

**DOI:** 10.1093/ckj/sfag091

**Published:** 2026-03-17

**Authors:** Rebecca Noble, Joanne Watt, Allister Irvine, Mary Jo Kurth, Peter Fitzgerald, Mark W Ruddock, Nicholas M Selby

**Affiliations:** Department of Renal Medicine, University Hospitals of Derby and Burton NHS Foundation Trust, Derby, DE22 3NE, UK; Centre for Kidney Research and Innovation, University of Nottingham, Nottingham, UK; Randox Laboratories Ltd, Crumlin, County Antrim, BT29 4QY, UK; Randox Laboratories Ltd, Crumlin, County Antrim, BT29 4QY, UK; Randox Laboratories Ltd, Crumlin, County Antrim, BT29 4QY, UK; Randox Laboratories Ltd, Crumlin, County Antrim, BT29 4QY, UK; Randox Laboratories Ltd, Crumlin, County Antrim, BT29 4QY, UK; Department of Renal Medicine, University Hospitals of Derby and Burton NHS Foundation Trust, Derby, DE22 3NE, UK; Centre for Kidney Research and Innovation, University of Nottingham, Nottingham, UK

**Keywords:** AKI, biomarkers, CKD, creatinine, cystatin C

## Abstract

**Background:**

Acute kidney injury (AKI) is common and associated with adverse long-term outcomes. Previously we have shown that a four-biomarker model of soluble tumour necrosis factor receptor-1 and -2 (sTNFR1, sTNFR2), cystatin C and estimated glomerular filtration rate (eGFR) measured 90 days after AKI performed well in predicting subsequent kidney disease progression. However, external validation in independent cohorts is essential to move these findings towards clinical application.

**Methods:**

A prospective, observational cohort of adults with AKI within 72 h of onset was assembled. Participants had study visits at time of AKI, then 30, 60 and 90 days later for biomarker sampling. Outcomes were assessed at 1 year, including major adverse kidney events (MAKE365, a composite of >25% decline in eGFR from baseline, kidney replacement therapy or death) and kidney disease progression. Logistic regression models incorporating biomarker combinations were evaluated using area under the receiver operating characteristic curve (AUC).

**Results:**

From 122 participants recruited at time of AKI, 89 survived and had biomarker measurements available from outpatient study visits. Of these, 35% developed MAKE365 and 30% had kidney disease progression at 1 year. The biomarker model (sTNFR1, sTNFR2, cystatin C, eGFR) measured at Day 90 discriminated those with MAKE365 with an AUC of 0.79 [95% confidence interval (CI) 0.68–0.91], and kidney disease progression with AUC 0.79 (95% CI 0.67–0.91). The biomarker model had comparable performance at earlier timepoints of Day 30 and 60.

**Conclusions:**

A biomarker panel comprising sTNFR1, sTNFR2, cystatin C and eGFR reliably predicts adverse outcomes up to 1 year post-AKI. This provides external validation of findings from previous biomarker discovery studies, and shows how this biomarker combination could be used to identify patients at lowest risk. This may support biomarker-guided approaches for personalized post-AKI risk stratification and follow-up.

## GRAPHICAL ABSTRACT

**GRAPHICAL ABSTRACT:**
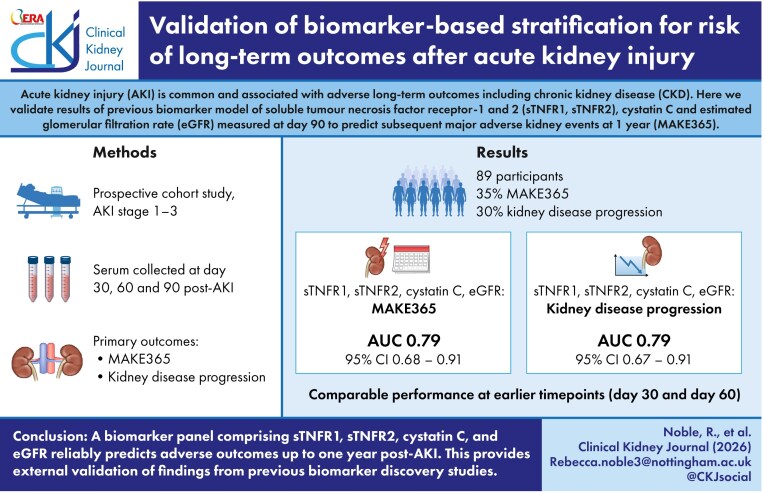

KEY LEARNING POINTS **What was known:**

- AKI is common and associated with adverse long-term outcomes.
- We have previously shown that a four-biomarker model of soluble tumour necrosis factor receptor-1 (sTNR1), sTNFR2, cystatin C and estimated glomerular filtration rate measured 90 days after AKI performed well in predicting subsequent kidney disease progression.

**This study adds:**

- This study validates this model in an external cohort, demonstrating that this combination of biomarkers reliably predicts adverse outcomes up to 1 year post–acute kidney injury (AKI).
- The biomarker model retains performance at earlier timepoints, i.e. Day 30 and 60 after AKI.
- Exploratory analysis showed that adding heart-type fatty acid binding protein (H-FABP) and midkine increased discriminatory performance.

**Potential impact:**

- This provides external validation of findings from previous biomarker discovery studies and support biomarker-guided approaches for personalized post-AKI risk stratification and follow-up.

## INTRODUCTION

Acute kidney injury (AKI) is a sudden loss of kidney function that occurs in up to 20% of hospitalized patients, affecting >13 million individuals worldwide each year [1] . AKI is associated with increased mortality and length of hospital stay [2]. Individuals who survive an episode of AKI are at elevated risk of long-term adverse outcomes, including the development or progression of chronic kidney disease (CKD) and reduced survival [3–5]. At present there are no proven diagnostic tools which can identify individuals who are at increased risk of long-term outcomes to assist in risk stratification.

Studies have reported associations between biomarker levels at time of AKI and risk of subsequent kidney disease progression [6, 7]. However, relatively few studies have investigated biomarkers in the post-AKI recovery period. Previously, we evaluated a panel of 11 biomarkers measured 90 days after AKI in the AKI Risk in Derby (ARID) study [8]. Using an unbiased approach, we identified soluble tumour necrosis factor receptor-1 (sTNFR1), soluble tumour necrosis factor receptor-2 (sTNFR2) and cystatin C, which in combination with estimated glomerular filtration rate (eGFR) were able to predict individuals at risk of kidney disease progression at 3 years [area under receiver operating characteristic curve (AUC) 0.79] [8]. Independently, Menez *et al*. used clinical and biomarker data measured 90 days after AKI from the Assessment, Serial Evaluation, and Subsequent Sequelae of AKI (ASSESS-AKI) study and also identified plasma cystatin C, sTNFR1 and sTNFR2 among the most promising predictor variables in models for subsequent major adverse kidney events (MAKE) and kidney disease progression [9]. A model that contained these three biomarkers, eGFR and four clinical variables was able to identify individuals at risk of a MAKE within 3 years after AKI with an AUC of 0.78. A combined analysis across ARID and ASSESS-AKI studies confirmed strong associations between sTNFR1 and sTNFR2 and subsequent mortality, heart failure and kidney disease progression [10]. Although promising, these results have not been validated in external cohorts, and the performance of these biomarker models at timepoints earlier than 3 months after an AKI event has not been studied.

To validate the performance of the biomarker models incorporating sTNFR1, sTNFR2 and cystatin C, measured 90 days after an episode of AKI, we investigated whether the biomarker combination was predictive for identifying individuals at risk of adverse outcomes in an independent, prospectively recruited cohort. Furthermore, we also investigated the relative performance of the biomarker combination at earlier post-discharge timepoints following AKI.

## MATERIALS AND METHODS

### Study design

A single-centre, prospective cohort study was performed (ClinicalTrials.gov registration: NCT05014022). The aim was to recruit an independent cohort to enable validation of the previously identified biomarker combinations (sTNFR1, sTNFR2, cystatin C) in discriminating individuals at elevated risk of kidney disease progression and mortality. Over 27 months, participants with AKI were recruited from a general inpatient hospital setting within 72 h of AKI onset. Participants were ≥18 years old, had AKI by creatinine-based KDIGO criteria [11], had at least one previous creatinine result available from the preceding 12 months for determining baseline kidney function, and were able to give informed consent. Baseline creatinine and eGFR were taken as most recent stable outpatient values preceding AKI. Exclusion criteria included obstructive uropathy, kidney transplant, patients receiving palliative care and suspected acute vasculitis or glomerulonephritis.

For each participant, data were collected detailing co-existing acute and long-term conditions, and Clinical Frailty Score [12]. Participants then had protocolized study visits at Days 30, 60 and 90 after AKI during which clinical information, blood and urine samples were collected. These visits were timed from onset of AKI rather than time of discharge. Participants received usual care during follow-up. Participants were followed up at 1 year after AKI onset with data collection for outcome assessment. eGFR was calculated using the 2009 Chronic Kidney Disease Epidemiology Collaboration creatinine equation without adjustment for ethnicity, in line with current UK guidance [13]. Ten millilitres of serum was collected from each participant at serial timepoints (time of AKI, Day 30, Day 60 and Day 90). Each sample was centrifuged at 3500 rpm for 15 min and stored in aliquots of 200 μL each per time point. Aliquots were stored at –20°C within 4 hrs of collection and then moved to –80°C within 7 days of initial collection. These samples were used for subsequently used for biomarker analysis.

### Biomarker analysis

Patient serum samples from Day 1, 30, 60 and 90 were analysed by Randox Clinical Laboratory Services (RCLS; Antrim, UK) using the kidney dysfunction biochip (sandwich chemiluminescent immunoassay) to measure sTNFR1 and sTNFR2 (Catalogue No. EV4560) (Randox Laboratories Ltd, Crumlin, UK). In addition to having an array of discrete test regions containing immobilized antibodies specific for sTNFR1 and sTNFR2, the kidney dysfunction biochip also has regions specific for heart-type fatty acid binding protein (H-FABP) and midkine. We therefore obtained results for all four biomarkers simultaneously. Using an Evidence MultiSTAT analyser (Catalogue No. EV4115) a light signal generated from each test region on the biochip is detected and the biomarker quantification is determined from a standard curve (Randox, Crumlin, UK). Cystatin C was measured on a RX Imola analyser (RX4900) according to manufacturer’s instructions (Randox, Crumlin, UK). This assay is standardized to certified reference material. Serum creatinine was measured using an enzymatic assay on the Roche Cobas 702 module (Roche Diagnostics Limited, West Sussex, UK). Our primary aim was to validate our previous biomarker combination of sTNFR1, sTNFR2, cystatin C and eGFR. In addition, we performed exploratory analyses that included midkine and H-FABP.

### Outcomes

Two main outcomes were chosen based on previous studies [8, 9]. The first was MAKE at Day 365 (MAKE365). This is a composite outcome of persistent kidney dysfunction (defined as a >25% drop in eGFR from baseline), need for kidney replacement therapy (KRT) or death. The second was kidney disease progression at Day 365 (defined as a >25% drop in eGFR from baseline, which includes individuals requiring ongoing KRT).

### Statistical analysis

Statistical analysis was performed in R (v4.4.2, R Foundation for Statistical Computing, Vienna, Austria) and SPSS (v29.0.2.0, IBM SPSS Statistics). Histograms were plotted for each data parameter to assess normality of the data. Participant characteristics are reported using mean ± standard deviation for parametric data and median [interquartile range (IQR)] for non-parametric data. Data were compared using the two-sided Wilcoxon rank sum test for non-parametric continuous data and using a Pearson’s Chi-squared test and Fisher’s exact test for categorical data using the gtsummary R package. A *P* < .05 was considered statistically significant. The Kruskal–Wallis test was used to compare biomarker values for the whole cohort over time. Where this was significant, Dunn’s test was applied. Correlations between biomarker concentrations and eGFR were assessed using the Pearson correlation coefficient.

Multivariable logistic regression was used to generate models to replicate those developed previously [8] and evaluate the performance of other biomarker combinations using area under the receiver operating characteristic curve (AUC) which were constructed using the pROC R package [14] . In line with previous studies, the Youden index was selected to select cut-off values as it provides a balanced measure of test performance by maximizing the sum of sensitivity and specificity.

### Missing data

Participants were recruited at the time of acute illness, so there was some loss to follow-up between the time of recruitment and first outpatient follow-up due to death during admission or before Day 30, in addition to expected losses to follow-up. To evaluate whether the missing outcome data introduced potential bias, a sensitivity analysis was performed that imputed missing Day 365 outcome data using the participants’ MAKE90 status.

## RESULTS

### Participant characteristics

**Figure 1:**
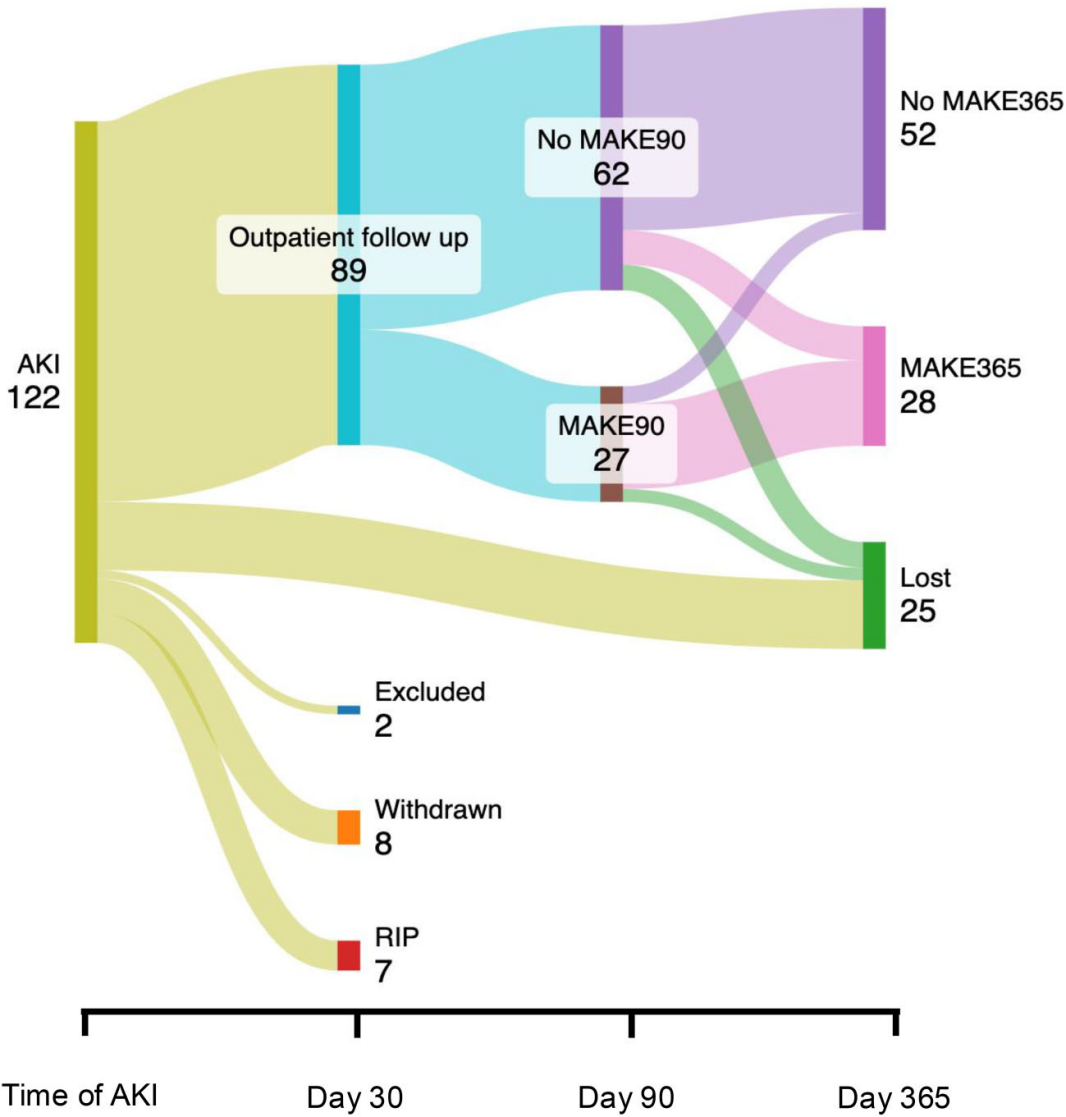
Sankey diagram of participant movement and outcomes over time. Of the nine participants lost between Day 90 and Day 365, none had died or was on RRT.

A total of 122 participants were recruited from a general inpatient hospital setting. Of these, 89 (73%) had at least one outpatient study visit (Day 30–Day 90) and provided samples for biomarker analyses. Participant flow through the study is described in the diagram in Fig. 1. Table 1 summarizes the key clinical characteristics of the 89 included participants. Over half the individuals (53, 60%) had AKI stage 3, followed by 20 with stage 2 (22%) and 16 with stage 1 (18%).

**Table 1:** Baseline characteristics and kidney parameters for overall study cohort and by MAKE365 outcome.

Characteristic	Study cohort (*n* = 89)	No MAKE365 (*n* = 52)	MAKE365 (*n* = 28)	*P*-value
Sex (M:F)	50:39 (56:44)	26:26 (50:50)	19:9 (68:32)	.12
Age (years)	64.3 ± 13.0	63.8 ± 14.5	65.9 ± 9.0	.85
Ethnicity				.27
White	86 (97)	50 (96)	27 (96)	
Asian	2 (2)	2 (4)	0	
Black	1 (1)	0	1 (4)	
Patient admitted from				.35
Home	87 (98)	52 (100)	27 (96)	
Residential home	1 (1)	0	1 (4)	
Nursing home	1 (1)	0	0	
Body mass index (kg/m^2^)	30.6 (26.0–34.6)	28.9 (24.8–33.5)	33.0 (29.0–36.3)	**.042**
Rockwood Frailty Score	2.9 ± 1.3	2.7 ± 1.1	3.5 ± 1.5	**.01**
Charlson comorbidity index	4.1 ± 2.3	3.6 ± 2.2	5.0 ± 1.9	**.008**
Comorbidities				
Hypertension	56 (63)	33 (63)	19 (68)	.69
Diabetes	37 (42)	20 (38)	15 (54)	.19
Ischaemic heart disease	31 (35)	17 (33)	11 (39)	.56
Heart failure	22 (25)	10 (19)	9 (32)	.20
Medication before admission				
ACEi/ARB	47 (53)	30 (58)	15 (54)	.72
NSAIDs	13 (15)	8 (15)	2 (7.1)	.48
Diuretics	24 (27)	13 (25)	9 (32)	.49
SGLT2i	1 (1)	0	1 (3.6)	.35
Beta blocker	23 (26)	10 (19)	10 (36)	.10
Calcium channel blocker	14 (16)	10 (19)	3 (11)	.53
New medication during admission				
ACEi/ARB	4 (4)	3 (5.8)	0	.55
NSAIDs	2 (2)	1 (1.9)	0	>.99
Diuretics	10 (11)	4 (7.7)	4 (14)	.44
SGLT2i	1 (1)	1 (1.9)	0	>.99
Baseline kidney function				
Creatinine (μmol/L)	83 (70–106)	83 (69–121)	93 (77–110)	.31
eGFR (mL/min/1.73 m^2^)	69 (52–89)	73 (45–90)	68 (59–84)	.43
CKD stage				.44
1–2	62 (70)	35 (67)	20 (71)	
3a	9 (10)	4 (8)	3 (11)	
3b	12 (13)	10 (19)	2 (7)	
4	6 (7)	3 (6)	3 (11)	
AKI detail				
Previous AKI in the last 12 months?	10 (11)	2 (3.8)	7 (25)	**.007**
AKI on admission	60 (67)	34 (65)	22 (79)	.22
Peak creatinine (μmol/L)	325 (198–559)	303 (184–455)	383 (242–578)	.17
Peak stage of AKI				.063
1	16 (18)	9 (17)	4 (14)	
2	20 (22)	17 (33)	3 (11)	
3	53 (60)	26 (50)	21 (75)	
Duration of AKI (days)	8 (3–32)	5 (2–13)	29 (9–90)	**<.001**
Required KRT	17 (19)	6 (12)	7 (25)	.20
Kidney biopsy	5 (6)	1 (1.9)	4 (15)	**.044**

Mean ± standard deviation, median (IQR) or *n* (%), are shown. *P*-values represent comparison between those with and without MAKE365 and were calculated by the two-sided Wilcoxon rank sum test for non-parametric continuous data and Pearson’s Chi-squared test and Fisher’s exact test for categorical data. A p value of <0.05 (in bold) was considered significant. ACEi, angiotensin-converting enzyme inhibitor; ARB, angiotensin-receptor blocker; NSAID, non-steroidal anti-inflammatory drug; SGLT2i, sodium-glucose cotransporter 2 inhibitor.

### Outcomes

At 1 year, 80 participants had available data of whom 28 (35%) had a MAKE365 outcome. This represented 6 (7.5%) who had died, 4 (5%) who were dependent on KRT and 18 (23%) who had a fall in eGFR of >25% from pre-AKI baseline. Figure 1 shows the proportions of participants categorized with a MAKE outcome at each of the different timepoints in the study. At 1 year, 22 (30%) of surviving participants were defined as having kidney disease progression.

### Description of biomarker profiles at serial timepoints after AKI

**Figure 2:**
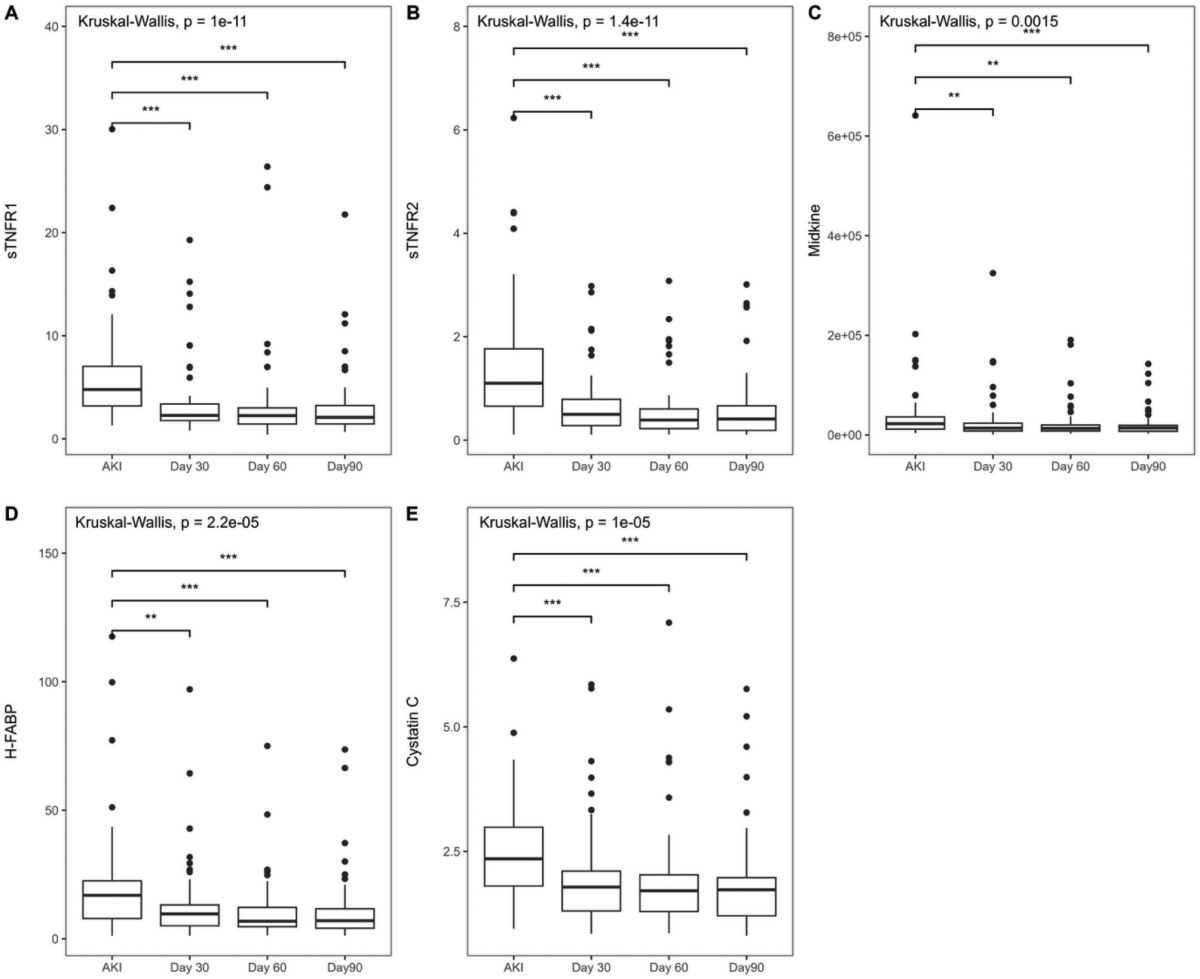
Box and whisker plots of biomarker values over time. Change over time compared using the Kruskal–Wallis test. This was significant for all biomarkers, and individual the timepoints were then compared using Dunn’s test, where: *P = .05; **P = .001; ***P < .001. Only timepoint comparisons that demonstrated statistically significances are shown.

Across all participants, median levels of sTNFR1, sTNFR2, midkine, H-FABP and cystatin C were higher at the time of AKI as compared with Day 30, with little change in any of the biomarkers between Days 30, 60 and 90 (Fig. 2, Supplementary data, Table S1). All five biomarkers correlated significantly with each other, as shown in Supplementary data, Fig. S1. There was an inverse relationship between the biomarkers and eGFR.

### Comparison of participants with and without MAKE365

When comparing those who did and did not experience MAKE at Day 365, there were no significant differences with respect to baseline kidney function, highest AKI stage or peak serum creatinine. A higher proportion of those with a MAKE365 outcome had experienced previous AKI in the 12 months prior to study entry [7 (25%) MAKE365 vs 2 (3.8%) no MAKE365, *P* = .007], and AKI duration was also longer in the MAKE365 group [29 days (9–90 days)] as compared with 5 days (2–13 days) in those without MAKE365 (*P* < .001). Those with a MAKE365 outcome had higher frailty (Clinical Frailty Score 3.5 ± 1.5 vs 2.7 ± 1.1, *P* = .01) and greater comorbidity (Charlson comorbidity index 5.0 ± 1.9 vs 3.6 ± 2.2, *P* = 0.008). Full characteristics of those who did and did not experience MAKE at Day 365 are shown in Table 1.

**Figure 3:**
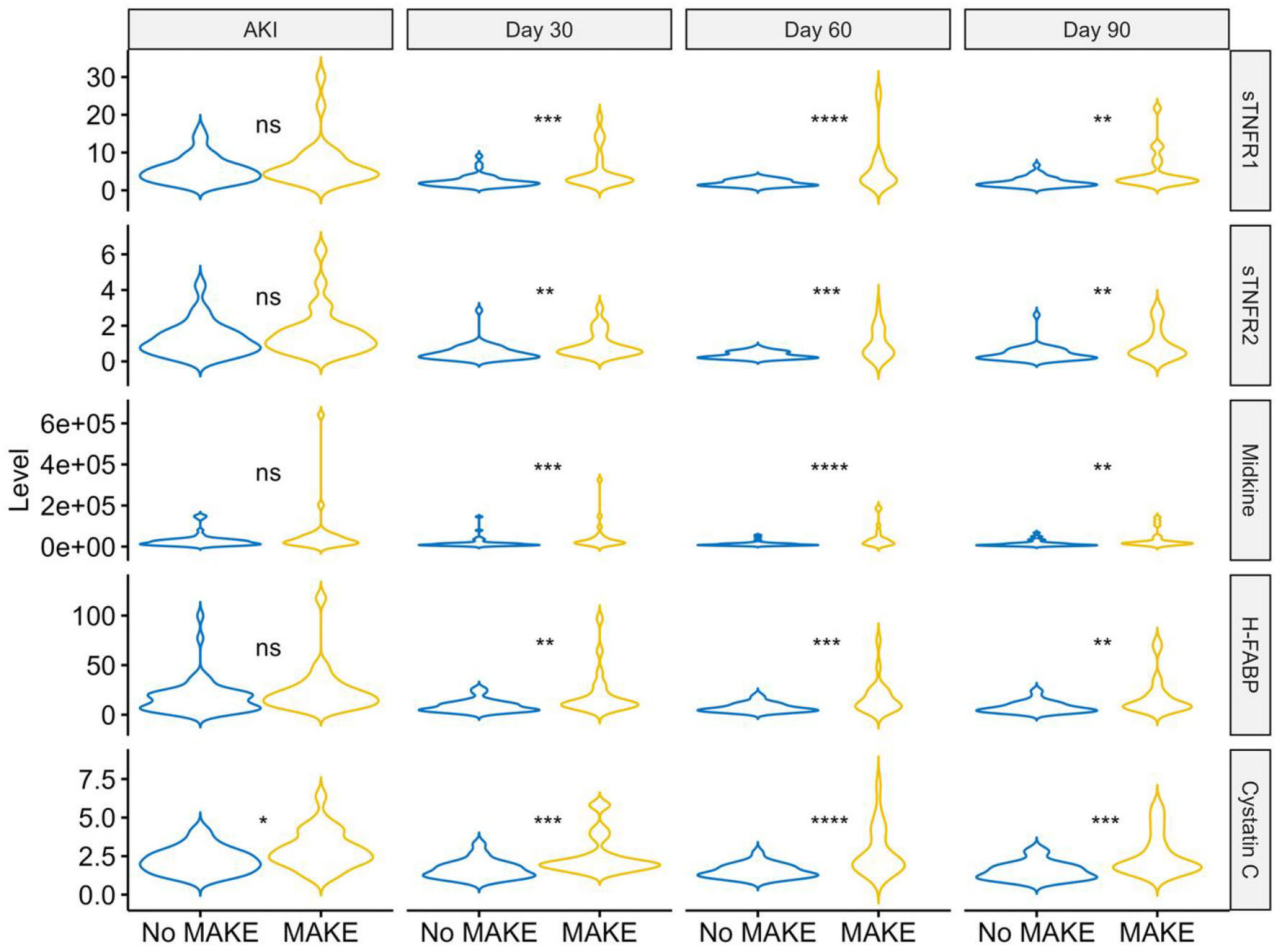
Violin plot of biomarker distribution at each timepoint separated into participants with and without MAKE outcome at 1 year, where: ‘ns’ is no significant difference; *P = .05; **P = .001; ***P < .001.

At the time of AKI, cystatin C was significantly higher in the MAKE365 group (median 2.61 mg/L, IQR 2.18–3.36 mg/L) as compared with those without (median 2.06 mg/L, IQR 1.56–2.81 mg/L, *P* = .013). There were no significant differences in any of the other biomarkers between groups. At all subsequent timepoints (Days 30, 60 and 90) cystatin C, sTNFR1, sTNFR2, H-FABP and midkine were all significantly higher in the MAKE365 group, as shown in Fig. 3 and Supplementary data, Table S2.

### Biomarkers at 90 days after AKI to predict outcomes at 1 year

**Figure 4:**
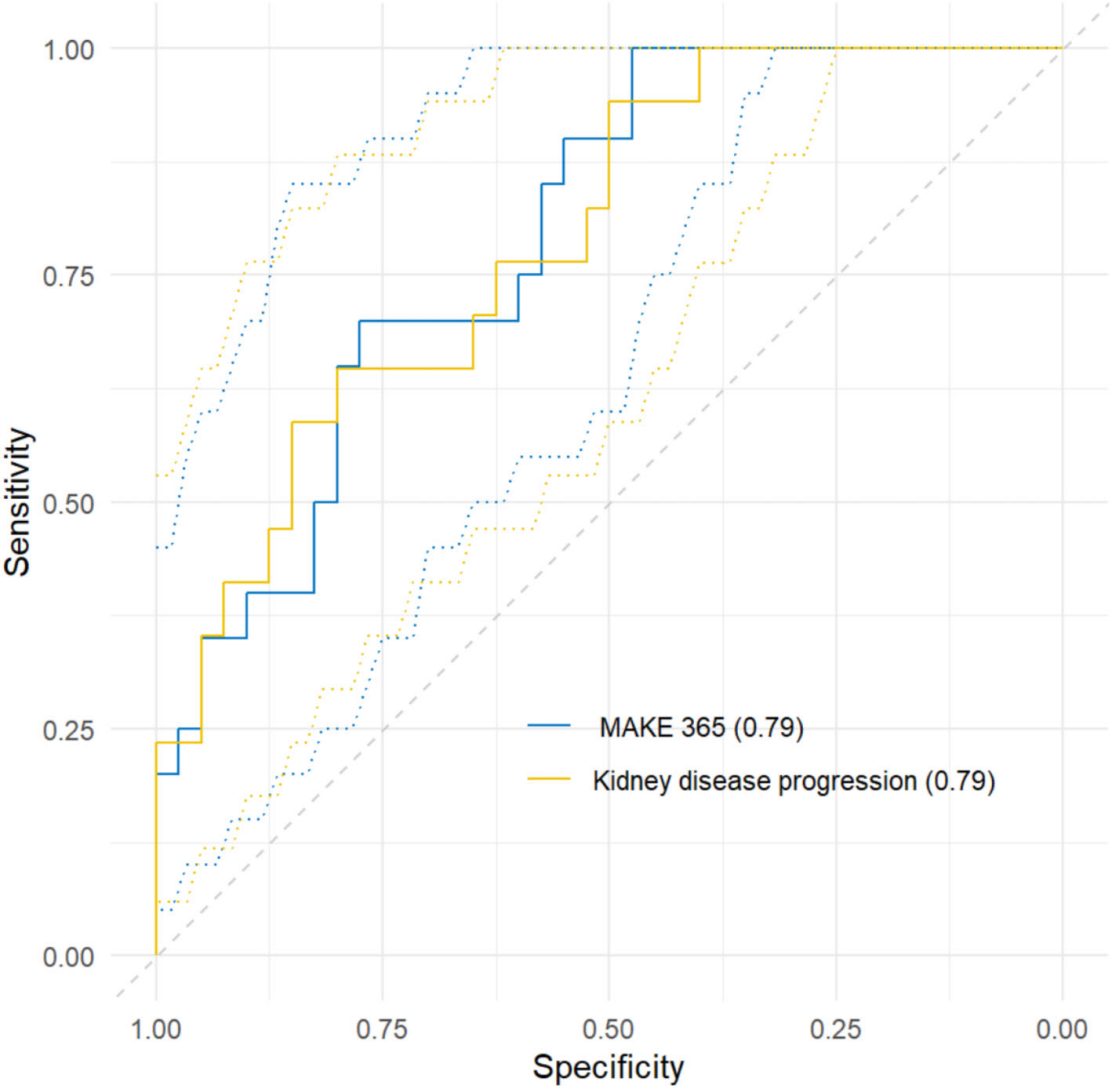
AUC for four-biomarker model with the outcomes of MAKE365 (blue) (AUC 0.79) and kidney disease progression (yellow) (AUC 0.79).

**Figure 5:**
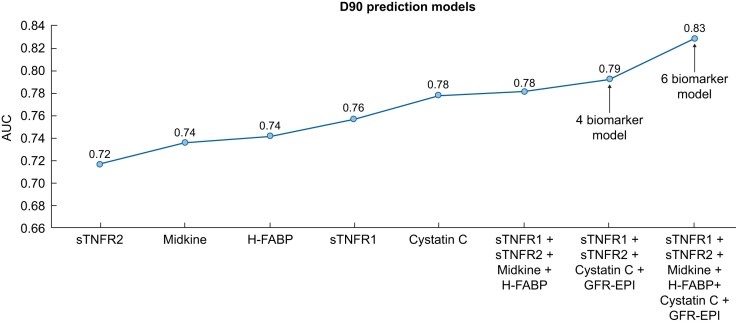
Plot of AUC values for individual and combination models including previous four-biomarker model [8] and a six-biomarker model including midkine and H-FABP. AUC for albumin:creatinine ratio was 0.5.

To validate findings from previous studies in this independent cohort, we firstly examined a four-biomarker combination of sTNFR1, sTNFR2, cystatin C and eGFR at Day 90 to predict MAKE365. This combination was able to discriminate individuals with and without MAKE365 outcomes with an AUC of 0.79 [95% confidence interval (CI) 0.68–0.91] as shown in Fig. 4. With a cut-off of –1.66, sensitivity was 100%, specificity 48%, positive predictive value (PPV) 49% and negative predictive value (NPV) 100%. The model had a higher AUC value than each individual biomarker in isolation as shown in Fig. 5. Sensitivity analysis using imputed data for the nine participants who were lost to follow-up did not show any significant change in AUC when they were included as per their status at Day 90 (AUC 0.79). We performed the same analyses with kidney disease progression at 1 year as the outcome. The model performed similarly with an AUC of 0.79 (95% CI 0.67–0.91). The model had a sensitivity of 65%, specificity of 80%, PPV of 58% and NPV of 84%. These models are summarized in Table 2.

**Table 2:** AUC analysis using a combination of sTNFR1, sTNFR2, cystatin C and eGFR at Day 90 to predict outcomes of MAKE and kidney disease progression at 1 year.

Outcome	AUC	95% CI	Cutoff	Sensitivity	Specificity	PPV	NPV
MAKE365	**0.79**	**0.68–0.91**	**–1.66**	**1.00**	**0.48**	**0.49**	**1.00**
Kidney disease progression at 1 year	**0.79**	**0.67–0.91**	**–0.62**	**0.65**	**0.80**	**0.58**	**0.84**
MAKE365 with imputed outcomes for 9 participants lost to follow-up	0.79	0.68–0.90	–1.16	0.91	0.57	0.54	0.92

A sensitivity analysis was performed in which missing data were imputed as per participant status at MAKE90 outcome.

### Biomarker performance at timepoints prior to Day 90

We then explored the performance of same biomarker combination of sTNFR1, sTNFR2, cystatin C and eGFR at earlier timepoints (Day 30 and Day 60) to predict MAKE365. This generated AUC values at Day 30 of 0.75 (95% CI 0.64–0.87) and at Day 60 of 0.83 (95% CI 0.74–0.93) (Supplementary data, Table S3).

### Exploratory analyses with additional biomarkers

In exploratory analyses, the performance of the additional biomarkers H-FABP and midkine on the kidney dysfunction biochip was assessed. At Day 90, the addition of H-FABP and midkine to the model containing sTNFR1, sTNFR2, eGFR and cystatin C (i.e. a six-biomarker combination) resulted in a numerically higher AUC of 0.83 (95% CI 0.72–0.92) with a higher specificity (83%) and PPV (67%) to identify individuals with MAKE365. Full results of these models and individual biomarker performance are included in Supplementary data, Table S3.

## DISCUSSION

The current study provides an independent validation of a previously reported four-biomarker model consisting of sTNFR1, sTNFR2, cystatin C and eGFR measured 90 days after AKI to determine risk of long-term adverse outcomes after AKI. We also demonstrate that the biomarker model retained performance at earlier timepoints, suggesting that this approach to evaluating patients’ long-term risk can be used across a wider time interval in the recovery period after AKI. The results suggest the biomarker combination will be most useful in identifying those at low risk of long-term outcomes after AKI. We also conducted exploratory analyses including H-FABP and midkine, showing potential utility of these biomarkers in this setting.

Despite the well-established link between AKI and CKD, a significant gap exists in the current provision of post-AKI follow-up care [3, 5]. Identifying individuals at highest risk of adverse outcomes following an episode of AKI could facilitate targeted follow-up and expand opportunities for proactive care. Some biomarkers measured at the time of AKI are associated with longer-term outcomes after AKI [7, 15–18]. However, there are inconsistencies in study design and short follow-up timeframes which limit the value of these results. Clinical risk scores that incorporate demographic and laboratory data exist, but have not yet translated into clinical practice [19]. For these reasons, there is interest in the identification of biomarkers that can be measured during the recovery period from AKI to better determine the risk of longer-term outcomes. Previously, we described the derivation and performance of a four-biomarker model combining sTNFR1, sTNFR2, cystatin C and eGFR in the ARID study, a prospective general AKI cohort from the UK. The biomarker model was able to discriminate at Day 90 between individuals with and without kidney disease progression at 3 years (AUC 0.79), with a high NPV that suggested clinical utility as a ‘rule-out’ test for identifying those individuals at low risk of kidney disease progression [8]. Recently, the US-based ASSESS-AKI study independently reported that a model containing the same four biomarkers (sTNFR1, sTNFR2, cystatin C and eGFR) and four other variables was able to discriminate those with MAKE within 3 years with an AUC of 0.78 [9]. Although promising, both studies concluded that these models required validation in separate populations before subsequent translation to clinical practice. We have addressed this in an independent, prospectively recruited cohort with longitudinal follow-up. Our results show that the performance of sTNFR1, sTNFR2, cystatin C and eGFR to discriminate MAKE365 and kidney disease progression at 1 year was similar to the original studies in which they were derived, with AUC values of 0.79 that suggest utility in clinical decision making [8, 9] (see also, Supplementary data, Table S4). This adds to the growing number of studies that have reported associations between sTNFR1 and sTNFR2 and subsequent kidney disease progression and other adverse outcomes including mortality in CKD, diabetic kidney disease, COVID-19 and post-surgical cohorts [7, 10, 20–24].

Similar to previous studies, we confirmed that a combination of biomarkers performed better than any of the individual biomarkers in isolation. It was also notable that there were relatively few differences in clinical parameters, including albuminuria, between those with and without adverse outcomes at 1 year that could be used to separate those at higher risk. We also observed the same high sensitivity and high NPV values as previously reported for both MAKE and kidney disease progression at 1 year, which suggests the biomarker combination will be of most use to identify low-risk patients who can avoid unnecessary and costly follow-up and monitoring. The clinical value of identifying lower risk participants is apparent when considering the large number of people who sustain and survive an episode of AKI each year, with estimates of 1.8 million people every year in high income countries alone [25]. Accepting that post-AKI follow-up is not possible for such large numbers, new approaches to tailor follow-up and management based on individual risk are therefore urgently needed. Adding to previous work, we also evaluated the performance of the four-biomarker model at serial timepoints between the time of AKI and Day 90. The discrimination was numerically lower at Day 30 and higher at Day 60, and although further work would be necessary to provide definitive comparisons across these different timepoints, it is reasonable to conclude that the four-biomarker model broadly retains performance over these earlier timepoints. This suggests that risk assessment could feasibly take place earlier during the recovery period from AKI, which may allow more flexibility with integration into clinical workflows in the future. Using the panel as a rule-out test would provide an opportunity to streamline services by reducing unnecessary follow-up for those at low risk of adverse outcome, as well as increasing opportunities for earlier intervention in those at higher risk.

We chose to measure four biomarkers of interest using a commercially available biochip (multiplex immunoassay) and an automated benchtop analyser. Although the immunoassay is currently available for research use only, the assay is well positioned in terms of its development for future clinical adoption. An ancillary benefit was that the kidney dysfunction biochip used to measure sTNFR1 and sTNFR2 also included additional biomarkers, H-FABP and midkine, allowing exploratory analyses. This combination of biomarkers has been shown to be predictive of AKI after cardiac surgery and in orthopaedic trauma patients undergoing open hip surgery [26, 27]. It has also been shown to predict progression of CKD caused by type 2 diabetes [28].

The addition of H-FABP and midkine to the other biomarkers increased the AUC value of the model, supporting future evaluation of this biomarker combination in a post-AKI setting.

Strengths of our study include its prospective nature that allowed independent validation of previous biomarkers in an independent cohort. Study design included protocolized assessments at serial timepoints between the time of AKI and 90 days after AKI, which is novel and allowed descriptions of the biomarker profiles over time. We also included MAKE and kidney disease progression as clinically relevant outcomes against which biomarkers were evaluated, matching the ARID and ASSESS biomarker studies. Our study also had limitations. The sample size was modest and was recruited from a single centre. Follow-up was shorter than the ARID and ASSESS biomarker studies, but can be justified as the rates of kidney progression were very similar at 1 year and 3 years in the main ARID cohort [29]. There were nine participants who were lost to follow-up after Day 90, although the sensitivity analysis with imputed outcomes for these participants did not change results significantly. Given that patients who died or were on KRT would have a known outcome it is highly probable that the participants who did not submit blood tests were alive and that this imputation is representative.

In summary, our results show that a four-biomarker model comprising sTNFR1, sTNFR2, cystatin C and eGFR retains performance in an independent cohort to assess risk of long-term MAKE and kidney disease progression after AKI. This supports future work in which this biomarker model (that has particular value in identifying low risk patients as a ‘rule-out’ test) is studied in combination with interventions targeted to improve outcomes after AKI or in the design of new pathways of care based on individual risk assessment and targeted follow-up.

## Supplementary Material

sfag091_Supplemental_Files

## Data Availability

Data available on request.
