# Supplementary material for: Validation of biomarker-based stratification for risk of long-term outcomes after acute kidney injury
Source: Clin Kidney J. 2026 Mar 17;19(5):sfag091. doi: 10.1093/ckj/sfag091 (PMC13139772; doi:10.1093/ckj/sfag091)
Supplement: sfag091_Supplemental_Files [file sfag091_supplemental_files.zip › Randox_AKI_supplementarymaterial_revision.docx]

**Supplementary Material**

|  | **Upper limit of normal** | **Time of AKI (n=115)** | **Day 30 (n=76)** | **Day 60 (n=69)** | **Day 90 (n=65)** |
| --- | --- | --- | --- | --- | --- |
| **sTNFR1 (ng/ml)** | 1.26 | 4.8 (3.1 – 7.1) | 2.21 (1.60 – 3.47) | 2.25 (1.41 – 2.92) | 2.08 (1.40 – 3.22) |
| **sTNFR2 (ng/ml)** | 0.29 | 1.15 (0.63 – 1.86) | 0.49 (0.28 – 0.81) | 0.39 (0.22 – 0.61) | 0.40 (0.18 – 0.68) |
| **Midkine (pg/ml)** | 9438 | 25034 (13290 – 37399) | 13215 (7658 – 24255) | 12267 (7759 – 19977) | 12550 (7025 – 18959) |
| **H-FABP (ng/ml)** | 5.50 | 17 (8 – 24) | 9.50 (4.94 – 13.70) | 6.98 (4.62 – 12.18) | 6.93 (4.27 – 11.62) |
| **Cystatin C (mg/l)** | 1.05 | 2.32 (1.78 – 3.12) | 1.77 (1.29 – 2.13) | 1.69 (1.32 – 2.03) | 1.72 (1.18 – 1.97) |
| **Serum creatinine (μmol/L)** | 84 | 325 (198 – 559) | 108 (84 – 163) | 99 (80 – 148) | 79 (103 – 143 |
| **eGFR (ml/min/1.73m^2^)** | >90 |  | 53 (34 – 82) | 57 (36 – 83) | 59 (35 – 81) |

**Supplementary table 1:** Biomarker values presented as median (interquartile range) for the whole cohort over time. Upper limit of normal is reported as per manufacturer instructions. Figure 2 presents these data as box-and-whisker plots and includes statistical comparisons between timepoints.

|  | **Time of AKI (n=76)** | | | **Day 30 (n=66)** | | | **Day 60 (n=63)** | | | **Day 90 (n=60)** | | |
| --- | --- | --- | --- | --- | --- | --- | --- | --- | --- | --- | --- | --- |
|  | **No MAKE 365** | **MAKE 365** | **P value** | **No MAKE 365** | **MAKE 365** | **P value** | **No MAKE 365** | **MAKE 365** | **P value** | **No MAKE 365** | **MAKE 365** | **P value** |
| **sTNFR1 (ng/ml)** | 4.8 (2.9 – 7.0) | 5.2 (3.7 – 8.6) | 0.429 | 2.00 (1.38 – 2.86) | 2.89 (2.30 – 6.92) | **<0.001** | 1.76 (1.22 – 2.61) | 2.92 (2.26 – 6.97) | **<0.001** | 1.86 (1.25 – 2.83) | 2.75 (2.26 – 5.40) | **0.001** |
| **sTNFR2 (ng/ml)** | 0.99 (0.63 – 1.75) | 1.15 (0.88 – 1.84) | 0.164 | 0.38 (0.20 – 0.67) | 0.66 (0.44 – 1.25) | **0.002** | 0.29 (0.20 – 0.52) | 0.65 (0.41 – 1.66) | **<0.001** | 0.29 (0.17 – 0.62) | 0.59 (0.31 – 1.24) | **0.007** |
| **Midkine (pg/ml)** | 20880 (10379 – 32093) | 28272 (14351 – 51214) | 0.144 | 11913 (7111 – 16371) | 23245 (13791 – 40289) | **<0.001** | 9288 (7033 – 14092) | 25691 (13418 – 37373) | **<0.001** | 9227 (5559 – 15875) | 18044 (12770 – 34053) | **0.003** |
| **H-FABP (ng/ml)** | 14 (6 – 22) | 18 (10-26) | 0.109 | 6.83 (4.36 – 11.29) | 11.55 (8.58 – 21.75) | **0.001** | 5.70 (3.68 – 9.91) | 10.52 (6.69 – 22.38) | **<0.001** | 5.97 (3.38 – 10.92) | 10.51 (7.02 – 20.28) | **0.003** |
| **Cystatin C (mg/l)** | 2.06 (1.56 – 2.81) | 2.61 (2.18 – 3.36) | **0.013** | 1.52 (1.19 – 1.98) | 2.05 (1.83 – 2.65) | **<0.001** | 1.42 (1.19 – 1.81) | 2.03 (1.87 – 3.58) | **<0.001** | 1.44 (1.07 – 1.89) | 1.93 (1.75 – 3.08) | **<0.001** |

**Supplementary Table 2:** Biomarker values over time by MAKE365 outcome, all data were non-parametric therefore presented as median (interquartile range). P value calculated using Wilcoxon signed-rank test. Values reported for all available data at each time point for those participants with 1 year outcome data

| **Model Formula** | **AUC** | **95% CI lower limit** | **95% CI upper limit** | **Patient Score Cutoff** | **Sensitivity** | **Specificity** | **PPV** | **NPV** |
| --- | --- | --- | --- | --- | --- | --- | --- | --- |
| D30 Cystatin C | 0.77 | 0.66 | 0.89 | -0.79 | 0.78 | 0.70 | 0.58 | 0.86 |
| D30 GFR-EPI | 0.71 | 0.59 | 0.82 | -0.24 | 0.57 | 0.75 | 0.55 | 0.77 |
| D30 ACR | 0.45 | 0.27 | 0.62 | -0.63 | 0.24 | 0.93 | 0.63 | 0.70 |
| D30 Midkine | 0.78 | 0.66 | 0.89 | -0.80 | 0.74 | 0.74 | 0.61 | 0.84 |
| D30 STNFR1 | 0.77 | 0.65 | 0.88 | -0.99 | 0.87 | 0.63 | 0.56 | 0.89 |
| D30 H-FABP | 0.74 | 0.62 | 0.86 | -1.00 | 0.87 | 0.56 | 0.51 | 0.89 |
| D30 STNFR2 | 0.74 | 0.62 | 0.86 | -0.99 | 0.83 | 0.56 | 0.50 | 0.86 |
| * D30 STNFR1 + STNFR2 + Cystatin C + GFR-EPI | 0.75 | 0.64 | 0.87 | -1.10 | 0.87 | 0.56 | 0.51 | 0.89 |
| ** D30 STNFR1 + STNFR2 + Midkine + H-FABP | 0.78 | 0.67 | 0.89 | -1.23 | 0.96 | -0.54 | 0.53 | 0.96 |
| ^#^D30 STNFR1 + STNFR2 + Midkine + H-FABP + Cystatin C + GFR-EPI | 0.76 | 0.64 | 0.88 | -1.29 | 0.96 | 0.47 | 0.49 | 0.95 |
| D60 Cystatin C | 0.84 | 0.73 | 0.94 | -0.72 | 0.76 | 0.81 | 0.67 | 0.87 |
| D60 GFR-EPI | 0.78 | 0.67 | 0.88 | -0.63 | 0.78 | 0.71 | 0.58 | 0.86 |
| D60 ACR | 0.54 | 0.37 | 0.72 | -0.91 | 0.35 | 0.80 | 0.43 | 0.74 |
| D60 Midkine | 0.80 | 0.67 | 0.92 | -0.89 | 0.67 | 0.83 | 0.67 | 0.83 |
| D60 STNFR1 | 0.81 | 0.70 | 0.92 | -1.22 | 0.91 | 0.62 | 0.54 | 0.93 |
| D60 H-FABP | 0.77 | 0.64 | 0.89 | -1.26 | 0.86 | 0.55 | 0.49 | 0.89 |
| D60 STNFR2 | 0.79 | 0.68 | 0.91 | -0.23 | 0.52 | 0.93 | 0.79 | 0.80 |
| * D60 STNFR1 + STNFR2 + Cystatin C D60 + GFR-EPI | 0.83 | 0.74 | 0.93 | -1.00 | 0.86 | 0.71 | 0.60 | 0.91 |
| ** D60 STNFR1 + STNFR2 + Midkine + H-FABP | 0.83 | 0.73 | 0.94 | -1.40 | 0.91 | 0.60 | 0.53 | 0.93 |
| ^#^D60 STNFR1 + STNFR2 + Midkine + H-FABP + Cystatin C D60 + GFR-EPI | 0.88 | 0.78 | 0.98 | -0.42 | 0.81 | 0.91 | 0.81 | 0.91 |
| D90 Cystatin C | 0.78 | 0.66 | 0.90 | -1.03 | 0.85 | 0.63 | 0.53 | 0.89 |
| D90 GFR-EPI | 0.79 | 0.68 | 0.89 | -0.51 | 0.74 | 0.75 | 0.61 | 0.84 |
| D90 ACR | 0.50 | 0.31 | 0.70 | -1.20 | 0.43 | 0.70 | 0.32 | 0.79 |
| D90 Midkine | 0.74 | 0.61 | 0.87 | -1.03 | 0.90 | 0.58 | 0.51 | 0.92 |
| D90 STNFR1 | 0.76 | 0.63 | 0.88 | -0.91 | 0.75 | 0.70 | 0.56 | 0.85 |
| D90 H-FABP | 0.74 | 0.62 | 0.87 | -1.09 | 0.80 | 0.60 | 0.50 | 0.86 |
| D90 STNFR2 | 0.72 | 0.58 | 0.85 | -1.12 | 0.90 | 0.48 | 0.46 | 0.91 |
| * D90 STNFR1 + STNFR2 + Cystatin C D90 + GFR-EPI | 0.79 | 0.68 | 0.91 | -1.66 | 1.00 | 0.48 | 0.49 | 1.00 |
| ** D90 STNFR1 + STNFR2 + Midkine + H-FABP | 0.78 | 0.66 | 0.90 | -0.46 | 0.65 | 0.83 | 0.65 | 0.83 |
| ^#^ D90 STNFR1 + STNFR2 + Midkine + H-FABP + Cystatin C + GFR-EPI | 0.83 | 0.72 | 0.93 | -0.38 | 0.70 | 0.83 | 0.67 | 0.85 |

**Supplementary table 3:** Individual and combination models to discriminate those with and without MAKE365, using cut-offs derived by Youden, * = 4 biomarker model (previous model) ** = kidney dysfunction biopchip ^#^ = 6 biomarker model. PPV (positive predictive value). NPV (negative predictive value).

| Study population | Outcome | Variables | AUC (95% CI) |
| --- | --- | --- | --- |
| ARID | Kidney disease progression at 3 years | sTNFR1, sTNFR2, cystatin C, eGFR_creat_ | 0.79 (0.70 – 0.83) |
| ASSESS-AKI | 3 year MAKE* | sTNFR1, plasma cystatin C, eGFR, BUN, FGF-23, sTNFR2, NT-ProBNP, UACR | 0.78 (0.66 – 0.90) |
| ASSESS-AKI | 3 year MAKE* | UACR, eGFR, urine YKL-40, sTNFR1 | 0.82 (0.68 – 0.96) |
| Current study | 1 year MAKE** | STNFR1 + STNFR2 + Cystatin C D90 + GFR-EPI | 0.79 (0.68 – 0.91) |
| Current study | 1 year MAKE** | D90 STNFR1 + STNFR2 + Midkine + H-FABP + Cystatin C + GFR-EPI | 0.83 (0.72 – 0.93) |

**Supplementary Table 4:** Comparison of previous study model combinations (ARID & ASSESS-AKI) with current study including outcomes and variables.

*MAKE defined as eGFR decline ≥ 40% or progression to ESKD within 3 years

**MAKE defined as eGFR decline ≥25% or progression to ESKD withing 1 year

**
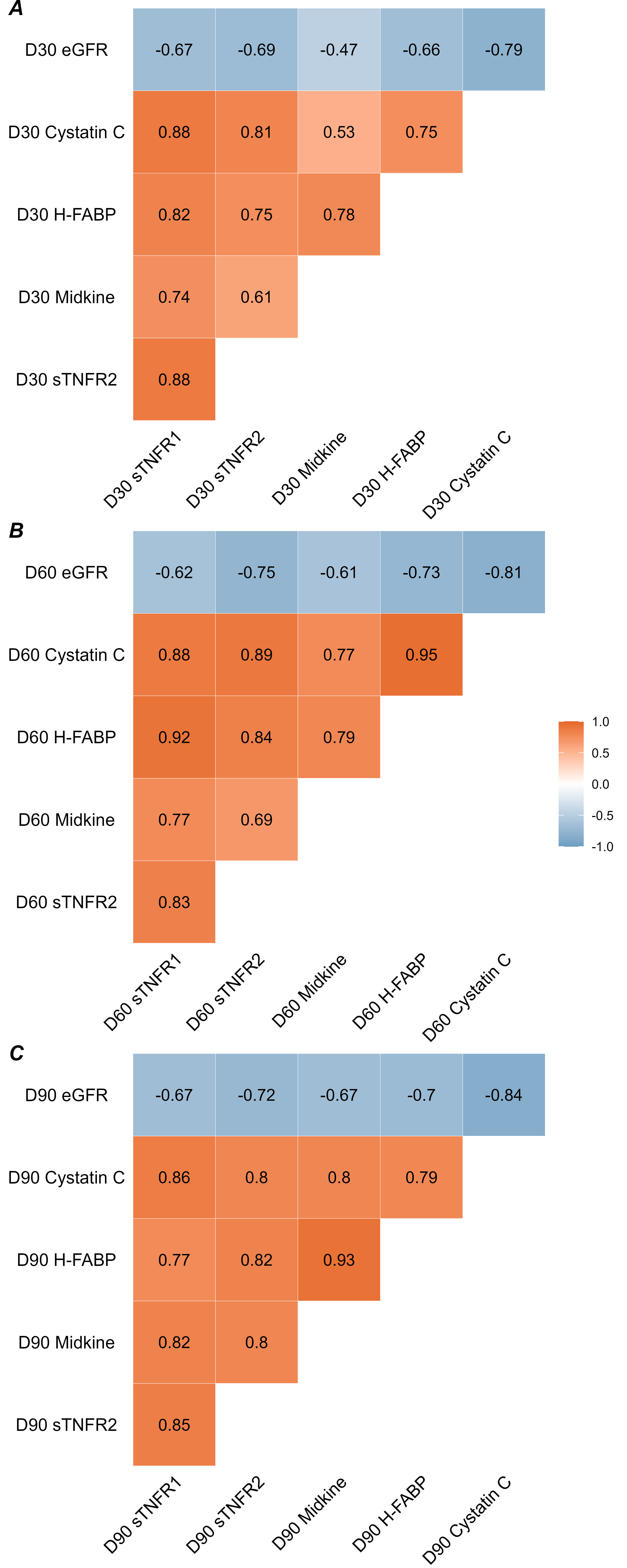
**

**Supplementary Figure 1:** Correlation matrices of biomarkers with each other and eGFR at the three follow-up timepoints (A = day 30; B = day 60, C = day 90), all correlations p<0.001
