# Supplementary material for: Validation of biomarker-based stratification for risk of long-term outcomes after acute kidney injury
Source: Clin Kidney J. 2026 Mar 17;19(5):sfag091. doi: 10.1093/ckj/sfag091 (PMC13139772; doi:10.1093/ckj/sfag091)
Supplement: sfag091_Supplemental_Files [file sfag091_supplemental_files.zip › Supplementary Figure 1_revision.pdf]

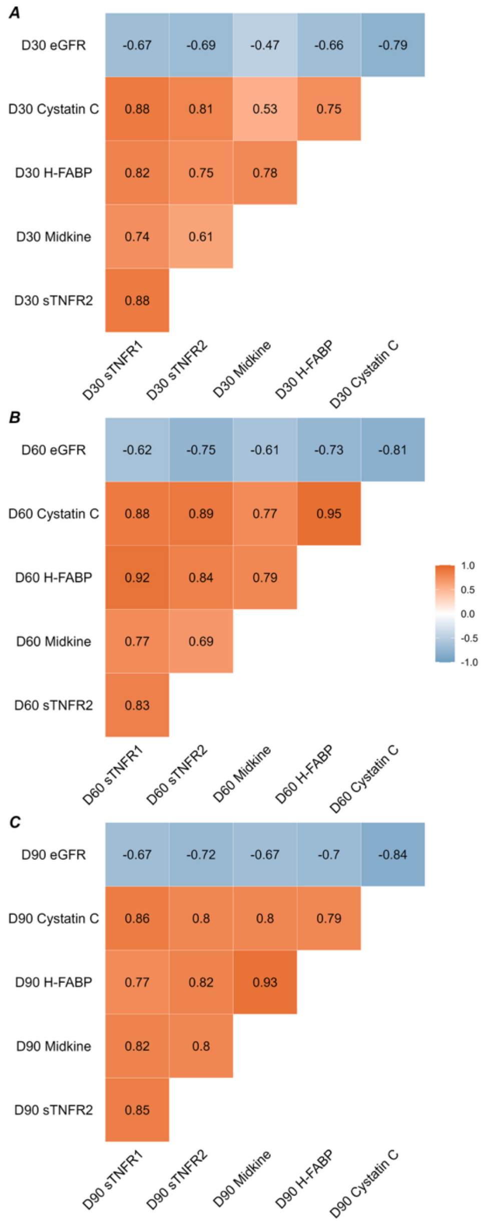

Supplementary Figure 1: Correlation matrices of biomarkers with each other and eGFR at the three follow-up timepoints (A = day 30; B = day 60, C = day 90), all correlations p<0.001
