# Supplementary material for: Validation of biomarker-based stratification for risk of long-term outcomes after acute kidney injury
Source: Clin Kidney J. 2026 Mar 17;19(5):sfag091. doi: 10.1093/ckj/sfag091 (PMC13139772; doi:10.1093/ckj/sfag091)
Supplement: sfag091_Supplemental_Files [file sfag091_supplemental_files.zip › Supplementary Table 1_revision.docx]

|  | **Upper limit of normal** | **Time of AKI (n=115)** | **Day 30 (n=76)** | **Day 60 (n=69)** | **Day 90 (n=65)** |
| --- | --- | --- | --- | --- | --- |
| **sTNFR1 (ng/ml)** | 1.26 | 4.8 (3.1 – 7.1) | 2.21 (1.60 – 3.47) | 2.25 (1.41 – 2.92) | 2.08 (1.40 – 3.22) |
| **sTNFR2 (ng/ml)** | 0.29 | 1.15 (0.63 – 1.86) | 0.49 (0.28 – 0.81) | 0.39 (0.22 – 0.61) | 0.40 (0.18 – 0.68) |
| **Midkine (pg/ml)** | 9438 | 25034 (13290 – 37399) | 13215 (7658 – 24255) | 12267 (7759 – 19977) | 12550 (7025 – 18959) |
| **H-FABP (ng/ml)** | 5.50 | 17 (8 – 24) | 9.50 (4.94 – 13.70) | 6.98 (4.62 – 12.18) | 6.93 (4.27 – 11.62) |
| **Cystatin C (mg/l)** | 1.05 | 2.32 (1.78 – 3.12) | 1.77 (1.29 – 2.13) | 1.69 (1.32 – 2.03) | 1.72 (1.18 – 1.97) |
| **Serum creatinine (μmol/L)** | 84 | 325 (198 – 559) | 108 (84 – 163) | 99 (80 – 148) | 79 (103 – 143 |
| **eGFR (ml/min/1.73m^2^)** | >90 |  | 53 (34 – 82) | 57 (36 – 83) | 59 (35 – 81) |

**Supplementary table 1:** Biomarker values presented as median (interquartile range) for the whole cohort over time. Upper limit of normal is reported as per manufacturer instructions. Figure 2 presents these data as box-and-whisker plots and includes statistical comparisons between timepoints.
