# Supplementary material for: Validation of biomarker-based stratification for risk of long-term outcomes after acute kidney injury
Source: Clin Kidney J. 2026 Mar 17;19(5):sfag091. doi: 10.1093/ckj/sfag091 (PMC13139772; doi:10.1093/ckj/sfag091)
Supplement: sfag091_Supplemental_Files [file sfag091_supplemental_files.zip › Supplementary Table 2_revision.docx]

|  | **Time of AKI (n=76)** | | | **Day 30 (n=66)** | | | **Day 60 (n=63)** | | | **Day 90 (n=60)** | | |
| --- | --- | --- | --- | --- | --- | --- | --- | --- | --- | --- | --- | --- |
|  | **No MAKE 365** | **MAKE 365** | **P value** | **No MAKE 365** | **MAKE 365** | **P value** | **No MAKE 365** | **MAKE 365** | **P value** | **No MAKE 365** | **MAKE 365** | **P value** |
| **sTNFR1 (ng/ml)** | 4.8 (2.9 – 7.0) | 5.2 (3.7 – 8.6) | 0.429 | 2.00 (1.38 – 2.86) | 2.89 (2.30 – 6.92) | **<0.001** | 1.76 (1.22 – 2.61) | 2.92 (2.26 – 6.97) | **<0.001** | 1.86 (1.25 – 2.83) | 2.75 (2.26 – 5.40) | **0.001** |
| **sTNFR2 (ng/ml)** | 0.99 (0.63 – 1.75) | 1.15 (0.88 – 1.84) | 0.164 | 0.38 (0.20 – 0.67) | 0.66 (0.44 – 1.25) | **0.002** | 0.29 (0.20 – 0.52) | 0.65 (0.41 – 1.66) | **<0.001** | 0.29 (0.17 – 0.62) | 0.59 (0.31 – 1.24) | **0.007** |
| **Midkine (pg/ml)** | 20880 (10379 – 32093) | 28272 (14351 – 51214) | 0.144 | 11913 (7111 – 16371) | 23245 (13791 – 40289) | **<0.001** | 9288 (7033 – 14092) | 25691 (13418 – 37373) | **<0.001** | 9227 (5559 – 15875) | 18044 (12770 – 34053) | **0.003** |
| **H-FABP (ng/ml)** | 14 (6 – 22) | 18 (10-26) | 0.109 | 6.83 (4.36 – 11.29) | 11.55 (8.58 – 21.75) | **0.001** | 5.70 (3.68 – 9.91) | 10.52 (6.69 – 22.38) | **<0.001** | 5.97 (3.38 – 10.92) | 10.51 (7.02 – 20.28) | **0.003** |
| **Cystatin C (mg/l)** | 2.06 (1.56 – 2.81) | 2.61 (2.18 – 3.36) | **0.013** | 1.52 (1.19 – 1.98) | 2.05 (1.83 – 2.65) | **<0.001** | 1.42 (1.19 – 1.81) | 2.03 (1.87 – 3.58) | **<0.001** | 1.44 (1.07 – 1.89) | 1.93 (1.75 – 3.08) | **<0.001** |

**Supplementary Table 2:** Biomarker values over time by MAKE365 outcome, all data were non-parametric therefore presented as median (interquartile range). P value calculated using Wilcoxon signed-rank test. Values reported for all available data at each time point for those participants with 1 year outcome data
