# Supplementary material for: Validation of biomarker-based stratification for risk of long-term outcomes after acute kidney injury
Source: Clin Kidney J. 2026 Mar 17;19(5):sfag091. doi: 10.1093/ckj/sfag091 (PMC13139772; doi:10.1093/ckj/sfag091)
Supplement: sfag091_Supplemental_Files [file sfag091_supplemental_files.zip › Supplementary Table 3_revision.docx]

| **Model Formula** | **AUC** | **95% CI lower limit** | **95% CI upper limit** | **Patient Score Cutoff** | **Sensitivity** | **Specificity** | **PPV** | **NPV** |
| --- | --- | --- | --- | --- | --- | --- | --- | --- |
| D30 Cystatin C | 0.77 | 0.66 | 0.89 | -0.79 | 0.78 | 0.70 | 0.58 | 0.86 |
| D30 GFR-EPI | 0.71 | 0.59 | 0.82 | -0.24 | 0.57 | 0.75 | 0.55 | 0.77 |
| D30 ACR | 0.45 | 0.27 | 0.62 | -0.63 | 0.24 | 0.93 | 0.63 | 0.70 |
| D30 Midkine | 0.78 | 0.66 | 0.89 | -0.80 | 0.74 | 0.74 | 0.61 | 0.84 |
| D30 STNFR1 | 0.77 | 0.65 | 0.88 | -0.99 | 0.87 | 0.63 | 0.56 | 0.89 |
| D30 H-FABP | 0.74 | 0.62 | 0.86 | -1.00 | 0.87 | 0.56 | 0.51 | 0.89 |
| D30 STNFR2 | 0.74 | 0.62 | 0.86 | -0.99 | 0.83 | 0.56 | 0.50 | 0.86 |
| * D30 STNFR1 + STNFR2 + Cystatin C + GFR-EPI | 0.75 | 0.64 | 0.87 | -1.10 | 0.87 | 0.56 | 0.51 | 0.89 |
| ** D30 STNFR1 + STNFR2 + Midkine + H-FABP | 0.78 | 0.67 | 0.89 | -1.23 | 0.96 | -0.54 | 0.53 | 0.96 |
| ^#^D30 STNFR1 + STNFR2 + Midkine + H-FABP + Cystatin C + GFR-EPI | 0.76 | 0.64 | 0.88 | -1.29 | 0.96 | 0.47 | 0.49 | 0.95 |
| D60 Cystatin C | 0.84 | 0.73 | 0.94 | -0.72 | 0.76 | 0.81 | 0.67 | 0.87 |
| D60 GFR-EPI | 0.78 | 0.67 | 0.88 | -0.63 | 0.78 | 0.71 | 0.58 | 0.86 |
| D60 ACR | 0.54 | 0.37 | 0.72 | -0.91 | 0.35 | 0.80 | 0.43 | 0.74 |
| D60 Midkine | 0.80 | 0.67 | 0.92 | -0.89 | 0.67 | 0.83 | 0.67 | 0.83 |
| D60 STNFR1 | 0.81 | 0.70 | 0.92 | -1.22 | 0.91 | 0.62 | 0.54 | 0.93 |
| D60 H-FABP | 0.77 | 0.64 | 0.89 | -1.26 | 0.86 | 0.55 | 0.49 | 0.89 |
| D60 STNFR2 | 0.79 | 0.68 | 0.91 | -0.23 | 0.52 | 0.93 | 0.79 | 0.80 |
| * D60 STNFR1 + STNFR2 + Cystatin C D60 + GFR-EPI | 0.83 | 0.74 | 0.93 | -1.00 | 0.86 | 0.71 | 0.60 | 0.91 |
| ** D60 STNFR1 + STNFR2 + Midkine + H-FABP | 0.83 | 0.73 | 0.94 | -1.40 | 0.91 | 0.60 | 0.53 | 0.93 |
| ^#^D60 STNFR1 + STNFR2 + Midkine + H-FABP + Cystatin C D60 + GFR-EPI | 0.88 | 0.78 | 0.98 | -0.42 | 0.81 | 0.91 | 0.81 | 0.91 |
| D90 Cystatin C | 0.78 | 0.66 | 0.90 | -1.03 | 0.85 | 0.63 | 0.53 | 0.89 |
| D90 GFR-EPI | 0.79 | 0.68 | 0.89 | -0.51 | 0.74 | 0.75 | 0.61 | 0.84 |
| D90 ACR | 0.50 | 0.31 | 0.70 | -1.20 | 0.43 | 0.70 | 0.32 | 0.79 |
| D90 Midkine | 0.74 | 0.61 | 0.87 | -1.03 | 0.90 | 0.58 | 0.51 | 0.92 |
| D90 STNFR1 | 0.76 | 0.63 | 0.88 | -0.91 | 0.75 | 0.70 | 0.56 | 0.85 |
| D90 H-FABP | 0.74 | 0.62 | 0.87 | -1.09 | 0.80 | 0.60 | 0.50 | 0.86 |
| D90 STNFR2 | 0.72 | 0.58 | 0.85 | -1.12 | 0.90 | 0.48 | 0.46 | 0.91 |
| * D90 STNFR1 + STNFR2 + Cystatin C D90 + GFR-EPI | 0.79 | 0.68 | 0.91 | -1.66 | 1.00 | 0.48 | 0.49 | 1.00 |
| ** D90 STNFR1 + STNFR2 + Midkine + H-FABP | 0.78 | 0.66 | 0.90 | -0.46 | 0.65 | 0.83 | 0.65 | 0.83 |
| ^#^ D90 STNFR1 + STNFR2 + Midkine + H-FABP + Cystatin C + GFR-EPI | 0.83 | 0.72 | 0.93 | -0.38 | 0.70 | 0.83 | 0.67 | 0.85 |

**Supplementary table 3:** Individual and combination models to discriminate those with and without MAKE365, using cut-offs derived by Youden, * = 4 biomarker model (previous model) ** = kidney dysfunction biopchip ^#^ = 6 biomarker model. PPV (positive predictive value). NPV (negative predictive value).
