# Supplementary material for: Validation of biomarker-based stratification for risk of long-term outcomes after acute kidney injury
Source: Clin Kidney J. 2026 Mar 17;19(5):sfag091. doi: 10.1093/ckj/sfag091 (PMC13139772; doi:10.1093/ckj/sfag091)
Supplement: sfag091_Supplemental_Files [file sfag091_supplemental_files.zip › Supplementary Table 4_new.docx]

| Study population | Outcome | Variables | AUC (95% CI) |
| --- | --- | --- | --- |
| ARID | Kidney disease progression at 3 years | sTNFR1, sTNFR2, cystatin C, eGFR_creat_ | 0.79 (0.70 – 0.83) |
| ASSESS-AKI | 3 year MAKE* | sTNFR1, plasma cystatin C, eGFR, BUN, FGF-23, sTNFR2, NT-ProBNP, UACR | 0.78 (0.66 – 0.90) |
| ASSESS-AKI | 3 year MAKE* | UACR, eGFR, urine YKL-40, sTNFR1 | 0.82 (0.68 – 0.96) |
| Current study | 1 year MAKE** | STNFR1 + STNFR2 + Cystatin C D90 + GFR-EPI | 0.79 (0.68 – 0.91) |
| Current study | 1 year MAKE** | D90 STNFR1 + STNFR2 + Midkine + H-FABP + Cystatin C + GFR-EPI | 0.83 (0.72 – 0.93) |

*MAKE defined as eGFR decline ≥ 40% or progression to ESKD within 3 years

**MAKE defined as eGFR decline ≥25% or progression to ESKD withing 1 year
